# Seasonality in malaria transmission: implications for case-management with long-acting artemisinin combination therapy in sub-Saharan Africa

**DOI:** 10.1186/s12936-015-0839-4

**Published:** 2015-08-19

**Authors:** Matthew E Cairns, Patrick G T Walker, Lucy C Okell, Jamie T Griffin, Tini Garske, Kwaku Poku Asante, Seth Owusu-Agyei, Diadier Diallo, Alassane Dicko, Badara Cisse, Brian M Greenwood, Daniel Chandramohan, Azra C Ghani, Paul J Milligan

**Affiliations:** MRC Tropical Epidemiology Group, LSHTM, London, UK; MRC Centre for Outbreak Analysis and Modelling, Imperial College London, London, UK; Kintampo Health Research Centre, Kintampo, Ghana; Faculty of Infectious and Tropical Diseases, LSHTM, London, UK; PATH-Malaria Vaccine Initiative, Dakar, Senegal; Malaria Research and Training Centre, Bamako, Mali; Université Cheikh Anta Diop, Dakar, Sénégal

**Keywords:** Malaria epidemiology, Seasonality, Heterogeneity, Artemisinin-based combination therapy, Post-treatment prophylaxis

## Abstract

**Background:**

Long-acting artemisinin-based combination therapy (LACT) offers the potential to prevent recurrent malaria attacks in highly exposed children. However, it is not clear where this advantage will be most important, and deployment of these drugs is not rationalized on this basis.

**Methods:**

To understand where post-treatment prophylaxis would be most beneficial, the relationship between seasonality, transmission intensity and the interval between malaria episodes was explored using data from six cohort studies in West Africa and an individual-based malaria transmission model. The total number of recurrent malaria cases per 1000 child-years at risk, and the fraction of the total annual burden that this represents were estimated for sub-Saharan Africa.

**Results:**

In settings where prevalence is less than 10 %, repeat malaria episodes constitute a small fraction of the total burden, and few repeat episodes occur within the window of protection provided by currently available drugs. However, in higher transmission settings, and particularly in high transmission settings with highly seasonal transmission, repeat malaria becomes increasingly important, with up to 20 % of the total clinical burden in children estimated to be due to repeat episodes within 4 weeks of a prior attack.

**Conclusion:**

At a given level of transmission intensity and annual incidence, the concentration of repeat malaria episodes in time, and consequently the protection from LACT is highest in the most seasonal areas. As a result, the degree of seasonality, in addition to the overall intensity of transmission, should be considered by policy makers when deciding between ACT that differ in their duration of post-treatment prophylaxis.

**Electronic supplementary material:**

The online version of this article (doi:10.1186/s12936-015-0839-4) contains supplementary material, which is available to authorized users.

## Background

The burden of malaria is not shared equally in endemic areas—some children experience repeated attacks of malaria while others remain healthy [1, 2]. Children who have multiple episodes of clinical malaria may be more likely to develop severe disease or severe anaemia [2–5], and recurrent malaria after discharge may lead to further severe morbidity in children who have been admitted to hospital [6, 7]. Repeated episodes of malaria may make children more susceptible to, or increase the severity of, other conditions such as invasive bacteraemia [8], and may also lead to poor school performance [9].

Artemisinin-based combination therapy (ACT) used for case management of malaria provides protection against a subsequent attack in the immediate period after treatment, because parasites that emerge from the liver while sufficient concentrations of the partner drug remain present in the blood will be killed. The duration of this post-treatment prophylaxis (PTP) depends on the pharmacokinetics and pharmacodynamics of the drug, as well as the resistance patterns in the parasite population to which individuals are exposed [10]. Thus, some repeat episodes of malaria will be prevented by use of any effective drug, but the number of episodes prevented will be greater where a longer-acting drug is used to manage the initial episode [11] and where the interval between attacks is short. Several long-acting forms of ACT (LACT) are now available, including dihydroartemisinin-piperaquine (DHA-PQ) and artesunate-mefloquine (AS-MQ), which provide several additional weeks of PTP compared to shorter-acting drugs. However, choice of first line treatment is not currently rationalized to exploit this benefit to its full potential.

**Fig. 1:**
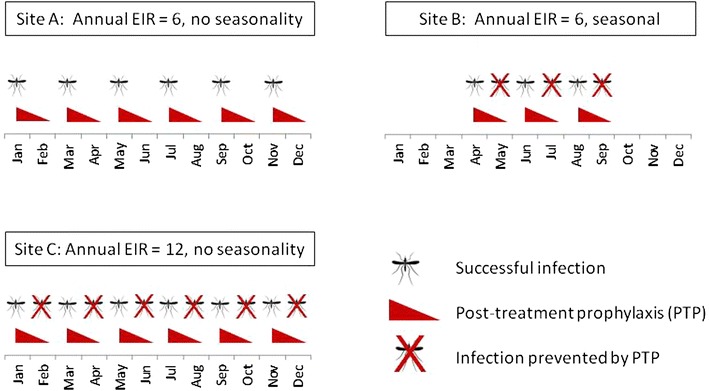
Illustration of the effect of seasonality on timing of malaria infections, and the benefit of post-treatment prophylaxis. *EIR* entomological inoculation rate, *PTP* post-treatment prophylaxis. Simplified representation of the effect of seasonality on timing of malaria infections. Sites A and B have the same annual EIR (6 infections per person per year). However, the monthly EIR in the seasonal setting (scenario B) is twice that in the perennial setting (scenario A). The monthly EIR during the transmission season in site B is the same as a perennial setting with an annual EIR of 12 (site C). Due to post-treatment prophylaxis provided by case-management for malaria, some malaria infections that occur soon after a previous episode will be prevented. This is more likely to occur where infection rates are higher, and where malaria infections occur close together in time. In seasonal settings, the length of PTP also becomes large in relation to the length of the season. This suggests that seasonal settings may have more preventable malaria than perennial settings with an equivalent annual EIR. Additional complications dealt with in this paper include realistic seasonality patterns, heterogeneity in malaria risk between individuals, immunity (acquisition of which may differ in seasonal and non-seasonal settings) and variable duration of protection from drugs used for case-management

The interval between malaria attacks will be short in high transmission settings, where reinfection is very common, until sufficient immunity is acquired to prevent disease. Cohort studies in highly endemic areas have shown a regular recurrence of symptomatic malaria, with intervals dependent on the drug used for treatment of the initial attacks [12–14]. A randomized trial in an area of high, seasonal transmission in Mali found that children who received artesunate plus a longer-acting partner drug (either amodiaquine or sulfadoxine-pyrimethamine) for each of their malaria episodes experienced fewer episodes over a 2 year period than children treated with the shorter-acting ACT, artemether-lumefantrine [11]. A recent model-based cost effectiveness study found that DHA-PQ would be more effective and cost-effective than artemether-lumefantrine in areas where transmission is high and seasonal [15]. The benefit of long-acting drugs may be greatest in high transmission, seasonal settings for a number of reasons. Firstly, areas with a high annual burden and a short season imply very high incidence rates (and very short intervals between episodes) during the season (Fig. 1). Secondly, in highly seasonal settings, a few weeks of PTP from a LACT may reflect a large proportion of the total time exposed each year: in the extreme case, in a setting with a transmission season 1 month in length, there should be no recurrent malaria if a drug providing more than 1 month of PTP is used for case-management. Finally, immunity may develop more slowly in seasonal settings due to gaps in exposure and decay in immune responses during the dry season.

However, to explore this further, and to generalize these findings to other drug combinations and to other interventions that could potentially be deployed to reduce the burden of repeated episodes of malaria, a better understanding of how seasonality and transmission intensity affect the epidemiology of repeated episodes of malaria is needed. This information could help inform policy-makers as to the likely importance of repeated episodes of malaria within a specific context, based on these underlying epidemiological drivers. In the present study, the timing and interval between repeat malaria episodes were estimated using data from six cohort studies in West Africa. An individual-based model of malaria transmission across sub-Saharan Africa was then used to explore the effects of transmission intensity and seasonality on the timing of repeat malaria across a range of epidemiological settings.

## Methods

### Data

Data on malaria incidence were analysed from six observational or experimental cohort studies in West Africa, conducted in Kintampo, Ghana [16]; Navrongo, Ghana [17]; Boussé, Burkina Faso [18, 19]; Kati district, Mali [20, 21]; Farafenni, The Gambia (Milligan, unpublished data) and Niakhar, Senegal [22]. Each of the field studies was approved by the Ethics Committee of the London School of Hygiene and Tropical Medicine and in the appropriate country, except for the Gambia cohort study which was approved by the MRC Gambia Ethics Committee. Further details of the studies and the sites are given in Table 1 and Additional file 1. In brief, these sites represent a range of transmission intensity, with cross-sectional prevalence ranging from 13 to 64 %, and a range of seasonality patterns, from year-round transmission in Kintampo, Ghana to highly seasonal transmission in the Gambia and Senegal. Genotyping was not undertaken to allow new infections to be distinguished from repeat attendances for the same clinical attack, or treatment failure, so to avoid double counting of the same clinical attack, malaria episodes within 7 days of a prior episode were not counted; this may exclude some genuine re-attendances for treatment failure. The seasonality in the incidence of malaria was then quantified, and the distribution of the intervals between malaria episodes examined as described below.

**Table 1:** Details of the cohort studies in West Africa

Author	Location	No. children^a^	No. malaria episodes^a^	Year of study	Drug used for case-management	Child age group	Transmission season	Prevalence	EIR	Insecticide-treated net coverage (%)
Cisse [22]	Niakhar, Senegal	744	1249	2002–2003	Chloroquine (first line), sulfadoxine-pyrimethamine (2nd line)	Under 5 years	August–October	2002: 36 % pre-season, 37 % post-season; 2003: 31 % pre-season, 30 % post-season	10 [45]	<1
Milligan (unpublished)	Farafenni, The Gambia	629	327	2003–2004	Chloroquine plus sulfadoxine-pyrimethamine	Under 10 years	August–October	post-season 2003: 38.7 % (under 10), 36.3 % (under 5)	10–40 (1997) [46]	1
Dicko [20, 21]	Kati district, Mali	1487	848	2008–2009	Artemether-lumefantrine	Under 5 years	August–November	13.2 % at end of transmission season	6.6–37.3 [21] (supplement S3)	99
Konate [18, 19]	Boussé district, Burkina Faso	1490	1097	2008–2009	Artemether-lumefantrine	Under 5 years	July–October	41.5 % at end of transmission season	173 [47]	93
Chandramohan [17]	Navrongo, Ghana	1141	710	2000–2004	Chloroquine	Infants	June–November	31.5 % at 18 months of age	418 [29]	3
Asante [16]	Kintampo, Ghana	1855	1600	2008–2011	Artesunate-amodiaquine	Infants	Perennial, peaks April–October	64 % (under 5) [48]	231–269 (2003–2005) [49]	~50

*EIR* entomological inoculation rate

^a^For intervention studies, this was based on the placebo group. Number of children and number of malaria episodes may not agree exactly with the published papers, due to restricting follow-up time in the present analyses to a single year of follow-up (to allow estimation of seasonality and the number of malaria episodes occurring per child per year), and discounting malaria episodes within 7 days of a prior episode

### Seasonality

The Markham seasonality index (MSI) [23] has previously been used to characterize malaria seasonality [24]. Full details are given in Additional file 2. In brief, the malaria incidence in each month is expressed as a vector, with a (different) fixed direction for each month, and with the length of the vector corresponding to the fraction of the annual incidence occurring in that month. Summation of the 12 monthly vectors gives a resultant vector, i.e. the finishing point back to the origin. The length of the resultant vector divided by the total length of all 12 monthly vectors gives the MSI (taking values ranging from 0, in the case where all months have equal incidence, and 1, in the case where all incidence occurs within 1 month). The direction of the resultant vector indicates the month in which the peak in incidence occurs. The MSI was calculated for each of the six studies.

### Timing of repeated episodes

The distribution of recurrent malaria episodes was inspected visually by constructing a histogram of the interval between episodes experienced by the same individual (based on date of first contact for each unique malaria episode). The mean interval between episodes was then obtained using the Kaplan–Meier estimate of the mean survival time among those who experienced a subsequent episode before the end of the follow-up period.

For the time varying hazard h(t), the survivor function for person treated on day x is

$$S\left( {x,T} \right) = { \exp }\left( { - \mathop \int \nolimits_{x}^{T} h\left( t \right)dt} \right)$$

where the time elapsed time from treatment is measured in days, x = 0 and T = 365.

The mean interval is then the area under the survival curve i.e.

$$\mathop \int \nolimits_{0}^{T} S\left( t \right)dt$$

and the average incidence over the year is the mean of h(t).

### Modelling

Having calculated these measures of seasonality and distribution of intervals between of repeat episodes for the six sites, an individual-based model (IBM) of *Plasmodium falciparum* described previously [25] was used to generalize these findings. This model describes the full transmission cycle of *P. falciparum* between humans and mosquitoes, as well as disease progression in humans, and has been fitted to extensive data on parasite prevalence determined by microscopy and polymerase chain reaction (PCR), and episodes of uncomplicated malaria by age and transmission setting across Africa [25, 26]. Heterogeneity in malaria risk is simulated by randomly assigning individuals at birth to experience different relative biting rates [25]. The model also incorporates the impact of insecticide-treated nets (ITNs). Anti-disease immunity, which reduces the probability of a blood-stage infection resulting in a clinical episode, is acquired at a rate that depends on exposure [25, 27]. The assumed parametric form of this immunity in the model gives rise to a lengthening of the interval between successive malaria attacks in areas of high endemicity, particularly among the most exposed sub-groups within a population who have higher exposure, and who therefore acquire protective immunity faster relative to their neighbours. The model has been fitted to age-incidence curves from 23 sites in Africa [26] but has not previously been compared to longitudinal patterns of repeat episodes. For this analysis, the timing of clinical episodes of malaria predicted by the model (i.e. when infection is symptomatic, and treatment is sought) were recorded for each individual in the simulation, to create a dataset of the timing of treated clinical episodes comparable to those from the six studies in West Africa.

Simulations were run using transmission and seasonality parameters appropriate to the first administrative unit (the largest sub-division within each country) within which the studies were located, to investigate if the model could approximately replicate the patterns seen in the six data sets in terms of seasonality and concentration of malaria episodes. Duration of post-treatment prophylaxis after treatment for malaria in each simulation was chosen to approximate that provided by the drug used in each study. Simulations were restricted to the same age group and with prevalence and levels of ITN coverage similar to those in the original studies.

### Model seasonality

Seasonality in transmission in the model is driven by temporal fluctuations in rainfall and temperature, which determine the ability of the environment to support development of mosquito larvae, referred to as larval carrying capacity (LCC) [28]. The LCC function was integrated by month for each of the 576 first administrative areas across Africa to estimate the MSI as described above, giving values of the MSI ranging from 1 to 91 %. However, all sites with an MSI <10 % were bimodal sites with two equal peaks spaced approximately 6 months apart. Nine sites were chosen representing each 10 % interval of the MSI between 10 and 90 % for further analysis, focusing on those without bimodal seasonality patterns, which were considered separately (see Additional file 3). To confirm that the seasonality in larval carrying capacity was reflected in incidence of clinical malaria predicted by the model, the Markham seasonality index was also calculated for symptomatic attacks of malaria predicted by the model in the nine sites.

### Modelling seasonality in different transmission intensity settings

For each of the seasonality patterns identified as described above (i.e. with MSI between 10 and 90 %), scenarios were simulated with mean annual parasite prevalence in 2–10 year old children of 5, 10, 20, 40 and 60 %, creating a 9 × 5 matrix of seasonality versus transmission intensity. For each of the 45 scenarios, all other parameters apart from the seasonality pattern and prevalence in 2–10 year olds were held constant. The set of parameters from the Upper East Region of Ghana (the first administrative unit in which the Navrongo study site is located) were used. This site was chosen because the epidemiology in this location has been well-characterized e.g. [29–35] and because this site was one of a number of sentinel sites used in assessing the fit of the model [25]. For all scenarios in the simulation, duration of post-treatment prophylaxis after case-management for malaria was assumed to be short (mean duration of 10 days).

Histograms showing the distribution of intervals between episodes were produced, and the estimated mean interval between malaria episodes calculated for each of the modelled scenarios using the Kaplan–Meier approach as described above. To understand the relative importance of seasonality and endemicity on the timing of repeat malaria episodes, the mean interval between episodes was plotted against the Markham seasonality index for each level of prevalence.

To explore the possible effect of post-treatment prophylaxis of varying duration, the model was used to estimate, for each scenario, the number of repeat malaria episodes that occur within 28, 42, 56 and 70 days of a previous attack, and the fraction of the annual burden that this represents. The result for the corresponding model scenario was then matched (in terms of seasonality and prevalence category) to indicate the absolute and relative importance of repeat malaria in these different periods, for each first administrative area in Africa.

## Results

### Data from cohort studies

In the cohort studies in West Africa, the seasonality in clinical malaria episodes increased moving from South to North, following the known patterns in seasonality of rainfall in the Sahel and sub-Sahel [36]. The Markham seasonality index (MSI), used to quantify seasonality in malaria incidence, was as follows: Kintampo, Ghana [16]: 42.8 %; Navrongo, Ghana [17]: 54.5 %; Boussé, Burkina Faso [18, 19]: 74.3 %; Kati, Mali [20, 21]: 82.3 %, Farafenni, The Gambia (Milligan, unpublished data): 85.5 %, Niakhar, Senegal [22]: 79.0 %. A graphical representation of the Markham seasonality index is shown for each site in Additional file  4.

**Fig. 2:**
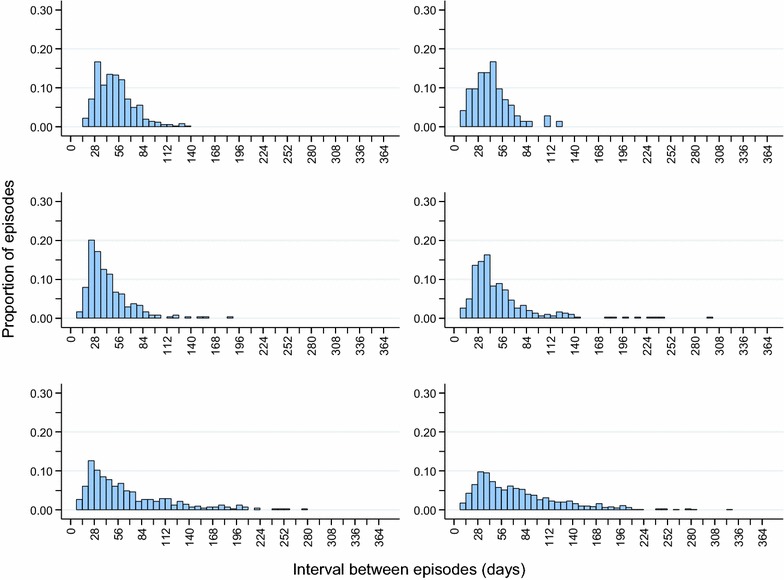
Distribution of intervals between repeat malaria episodes from 6 sites in West Africa. *Top row* Niakhar, Senegal, 2003; Farafenni, The Gambia, 2003; *Middle row* Kati, Mali 2008–2009; Boussé, Burkina Faso, 2008–2009. *Bottom row* Navrongo, Ghana 2002–2003; Kintampo, Ghana 2010–2011. The mean interval between malaria episodes, in days, was 50.9 (95 % CI 49.0, 52.8) days in Niakhar, 42.3 (95 % CI 37.0, 47.5) days in Farafenni, 43.4 (95 % CI 40.1, 46.7) days in Kati, 52.4 (95 % CI 48.0, 56.7) days in Boussé, 66.1 (95 % CI 60.6, 71.6) days in Navrongo, and 74.6 (95 % CI 71.1, 78.0) days in Kintampo

The distribution of the intervals between repeat episodes of malaria for each site is shown in Fig. 2. Moving from South to North (i.e. from less seasonal to more seasonal settings) the mean interval between episodes, estimated using the Kaplan–Meier method, decreased: mean interval, 74.6 (95 % CI 71.1, 78.0) days in Kintampo, 66.1 (95 % CI 60.6, 71.6) days in Navrongo, 52.4 (95 % CI 48.0, 56.7) days in Boussé, 43.4 (95 % CI 40.1, 46.7) days in Kati, 42.3 (95 % CI 37.0, 47.5) days in Farafenni, and 50.9 (95 % CI 49.0, 52.8) days in Niakhar.

**Fig. 3:**
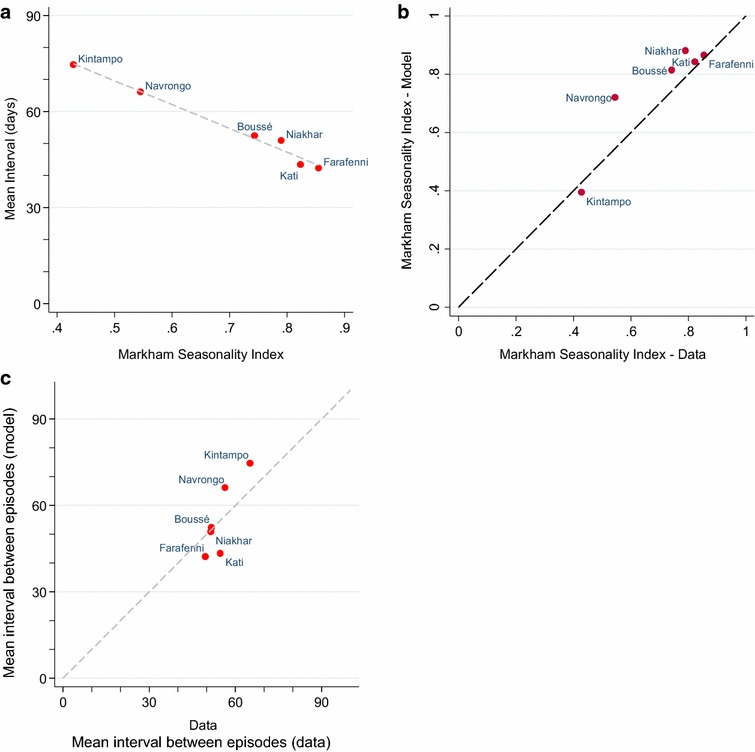
Markham seasonality index and interval between episodes, model fits to data. **a** The estimated mean interval between malaria episodes shown against the Markham seasonality index (as a measure of seasonality 0, not seasonal; 1, highly seasonal) for each of the six studies. The *grey dashed line* shows the line of best fit. **b** Markham seasonality index for each of the six studies in West Africa, against the MSI for the model-based seasonality pattern for the first administrative unit in which the site is located: Kintampo, Brong-Ahafo region; Navrongo, Upper East Region; Boussé, Kourwéogo; Kati, Koulikoro; Niakhar, Fatick; Farafenni, North Bank Division. **c** mean interval between malaria episodes (in days) from the six studies in West Africa, against the mean interval between episodes (in days) in model simulations for the first administrative unit in which the study site is located

A plot of the observed MSI against the mean interval between episodes is shown in Fig. 3. This suggests that the average interval between episodes decreases as seasonality increases, although interpretation of this based on the data alone is complicated by differences in transmission intensity, and other differences between the sites, including the use of drugs used for case-management. This was chloroquine or chloroquine plus sulfadoxine-pyrimethamine in the three earlier studies (long-acting but likely subject to some resistance) and an ACT in the three latter studies (highly effective but shorter-acting).

### Model simulations

**Fig. 4:**
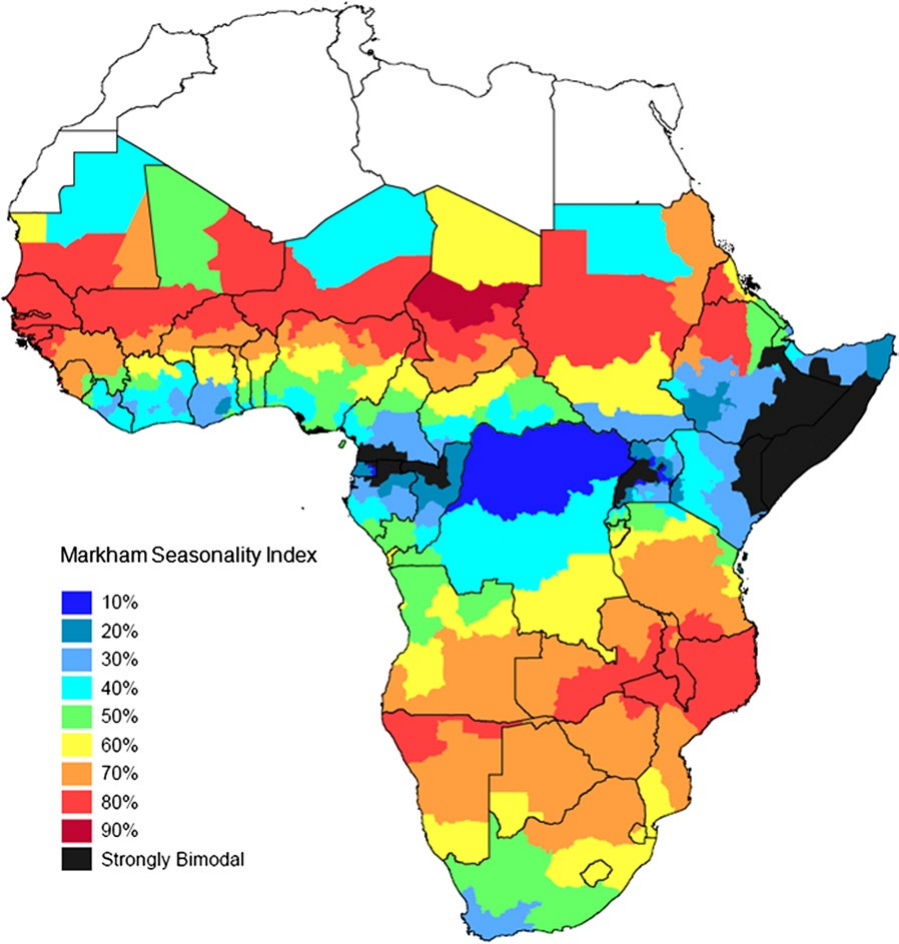
Markham seasonality index by first administrative area.* Figure* shows Markham seasonality index for larval carrying capacity (LCC) for each first administrative area (the largest administrative sub-division within country.* Black areas* show those with strongly bimodal seasonality patterns (see Additional file 3 for details)

The MSI of the larval carrying capacity (the suitability of the environment for development of malaria vectors [28]) is shown for each of the 576 first administrative areas in Africa simulated by the model in Fig. 4. Relatively few locations showed a strong degree of bimodality in malaria transmission; these are dealt with separately in Additional file 3 and summarized below. Seasonality profiles and the corresponding Markham seasonality polygons for the sites representing 10 % intervals of the MSI (from 10 to 90 %) are shown in Additional files 5, 6. The model predictions of the MSI was very close to the observed values for the locations in which the six studies were conducted (Fig. 3b). The correlation between seasonality in larval carrying capacity (LCC) input into the model and seasonality in malaria incidence predicted by the model was very strong at all levels of prevalence (Additional file 7). The mean interval between malaria episodes predicted by the model was also similar for the locations where the cohort studies were conducted (Fig. 3c).

**Fig. 5:**
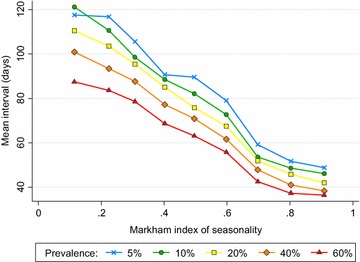
Model-predicted mean interval between malaria episodes versus Markham seasonality index. Mean interval between recurrent malaria episodes, according to seasonality as measured by the Markham Seasonality Index. Prevalence: modelled prevalence in 2–10 year olds 5, 10, 20, 40 and 60 %, as indicated

Figure 5 shows the estimated mean interval between episodes plotted against the Markham seasonality index for each of the 45 scenarios modelled (nine seasonality patterns (10–90 %), and five prevalence levels (5, 10, 20, 40 and 60 % in 2–10 year olds). The distribution of intervals between malaria episodes is shown in Additional file 8. Malaria episodes occur in closer succession in higher prevalence settings, where more infections occur per unit time. At a given prevalence, the average interval between repeat episodes decreases with increasing seasonality, and is always shortest in the most seasonal transmission settings. In particular, a large part of the burden of repeat malaria episodes occurs soon after a previous episode in highly seasonal, high prevalence areas. Bimodality increases the average interval between episodes compared to unimodal seasonal settings of similar endemicity, because there are two periods when cases are more likely to occur, separated by a period of lower risk (Additional file 3).

**Fig. 6:**
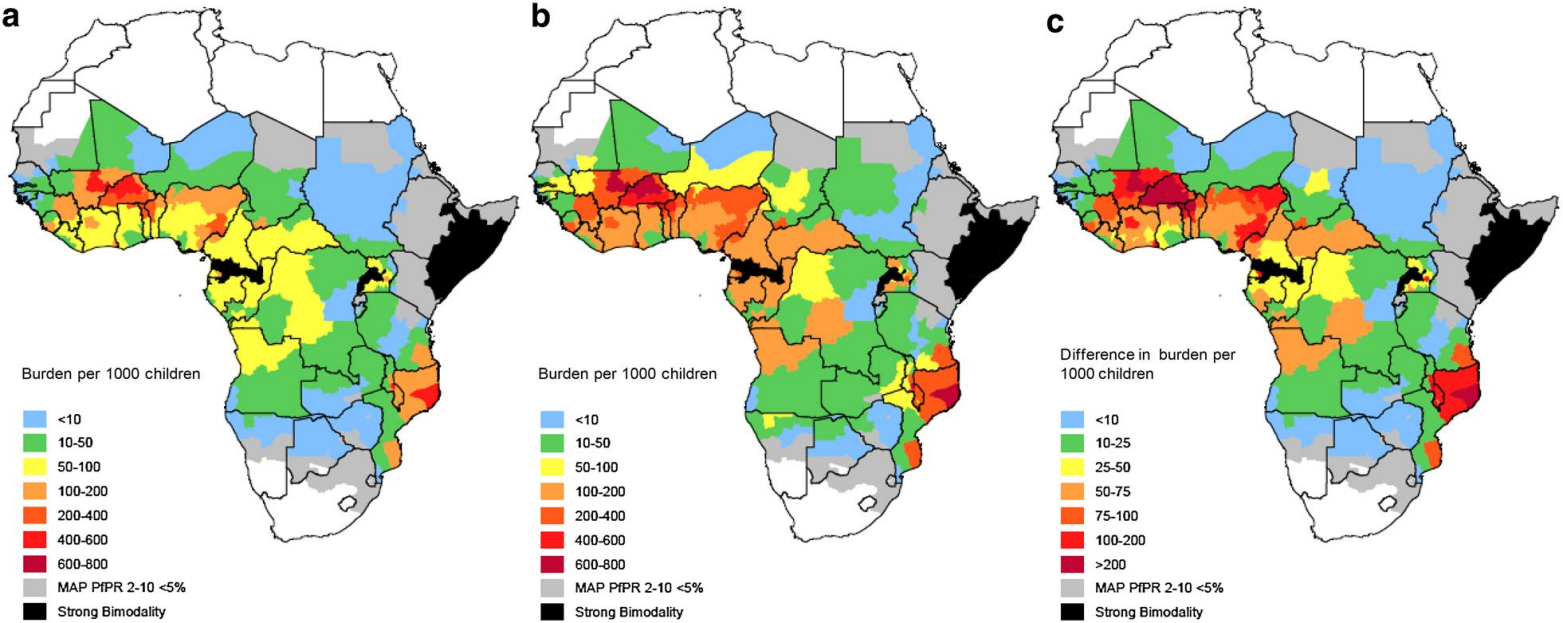
Burden of repeat malaria episodes per 1000 children by first administrative unit across Africa. Burden of malaria episodes per 1000 children that occur within **a** 28 days and **b** 42 of a prior episode. **c** shows the additional burden per 1000 children that occurs between 28 and 42 days after a previous episode

**Fig. 7:**
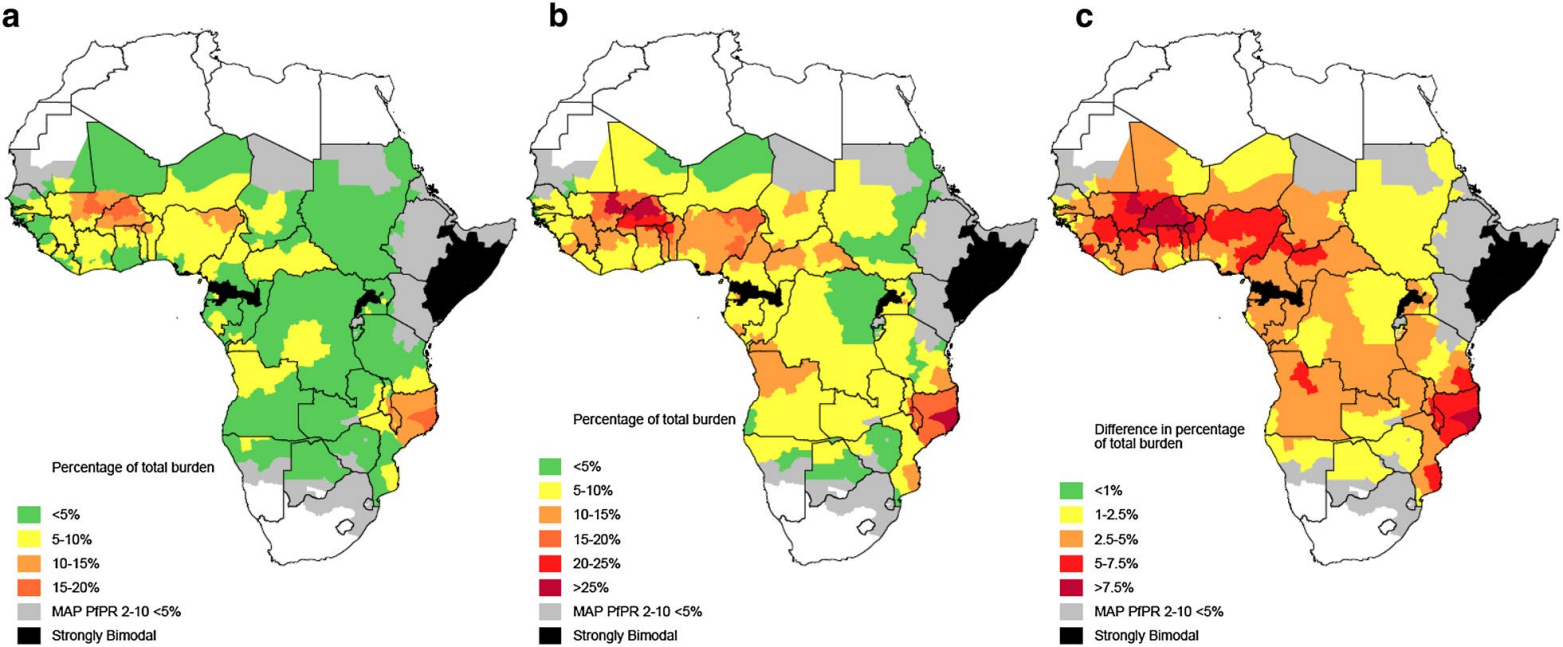
Percentage of malaria burden in children occurring within 28 and 42 days by first administrative unit across Africa. Fraction of all malaria episodes in children under 5 years of age predicted to occur within **a** 28 days and **b** 42 of a prior episode. **c** shows the difference in the overall fraction of episodes among children that occurs between 28 and 42 days after a previous episode

Figure 6 shows the burden of repeat malaria episodes within 28 and 42 days of a previous attack in absolute terms, as well as the incremental increase in the burden of repeat malaria between 28 and 42 days after the preceding episode. Figure 7 shows the burden of repeat malaria within 28 and 42 days as a fraction of the total malaria burden (all episodes, including first episodes) in children under 5 years of age. The highest burden of repeat malaria occurring soon after a previous episode appears to be in the Sahel and sub-Sahel regions of Africa, where malaria transmission is intense and highly seasonal. Repeat malaria also appears to be important in a seasonal focus encompassing parts of Malawi, Mozambique and Southern Tanzania, and in areas of perennial transmission with a very high burden in Eastern Uganda.

Interventions able to tackle the burden of repeat malaria soon after treatment are likely to have an important impact on malaria burden in highly endemic settings, and particularly in highly endemic and highly seasonal settings. In such settings, repeat malaria within 28 days of a previous episode constitutes up to 20 % of the overall malaria burden in children (Additional file 9). Within 42 days, this share of the overall burden rises to around 30 %. At 60 % prevalence, an estimated 250 cases per 1000 children per year occur within 42 days of a prior attack in perennial settings, rising to 1000 cases per 1000 children in highly seasonal settings (Additional file 10). In such areas, the incremental effect of a drug providing an additional 2 weeks of PTP (42 days versus 28 days) could prevent in excess of 200 cases per 1000 children, per year.

In areas with highly seasonal transmission and moderate-high transmission (e.g. 2–10 year old prevalence of 40 %), repeat malaria can, relative to non-seasonal settings with substantially higher prevalence (2–10 year old prevalence of 60 %), account for a higher number of cases (e.g. Additional file 10) and a higher proportion of the overall malaria burden (Additional file 9).

In settings with a prevalence in 2–10 year old children less than 20 %, repeat malaria is relatively unimportant. This is partly because there is a small absolute number of repeat episodes (Additional file 10), and partly because repeat episodes soon after a previous attack constitute a small fraction of the overall burden (Additional file 9).

While the burden within 56 and 70 days of a prior episode is larger than that within 28 and 42 days, the incremental increase in the burden with increasing time since the previous episode is progressively smaller as the post-treatment interval increases (Additional files 11, 12). In the most seasonal settings (MSI 90 %), these incremental values are smaller than in slightly less seasonal settings (MSI 70, 80 %). This is because the distribution of repeat malaria episodes peaks very sharply soon after a previous episode in extremely seasonal settings, and because malaria risk becomes very small when the post-episode window extends into the dry season. The advantage of LACTs with a longer mode of action than those currently available (e.g. an ACT that provided 10 weeks of PTP) may therefore be greater in settings where the distribution of repeat malaria episodes is spread slightly more evenly over time (i.e. high burden and seasonal, but not extremely seasonal). Other than where transmission is extremely seasonal, and particularly in perennial settings, longer PTP is always likely to be advantageous as although second events may occur close to the preceding episode in high transmission settings, subsequent episodes may also be prevented.

## Discussion

This analysis investigated the burden of repeated malaria episodes and the interval between successive episodes in cohort studies conducted in a range of epidemiological settings in West Africa, and used a model to estimate the effects of seasonality and transmission on the amount and timing of repeat malaria episodes across the continent. These results show that seasonality has an important effect on the distribution of recurrent malaria episodes, and should be considered alongside overall transmission intensity when deciding between different regimens for the case-management of malaria.

Repeated malaria attacks within a short time frame are not common where malaria transmission is low, and very few repeat episodes occur within the period of post-treatment prophylaxis provided by currently available drugs. This means that the additional benefit of a long-acting ACT over a shorter-acting drug in low transmission settings is likely to be minimal, and other factors such as cost, simplicity of dosing, tolerability and local resistance patterns to ACT partner drugs are likely to be more important considerations. Where malaria transmission is higher, repeated episodes of malaria are more common, and a larger fraction of the annual burden occurs within the period of post-treatment prophylaxis provided by longer-acting ACT, and therefore might be prevented if these regimens were used in place of shorter-acting regimens. In higher transmission settings, the burden is also larger in absolute terms, and the burden averted is also, therefore, likely to be larger.

At a given level of transmission intensity, the fraction of episodes occurring within a fixed time period after a previous attack increases in a non-linear fashion with increasing seasonality. This is plausible as in settings with a short season, any recurrent malaria must occur relatively close to a preceding episode, and because, to achieve a similar level of transmission in a shorter period, the transmission intensity must be higher during the season. Seasonality may also lead to more recurrent malaria because immunity is not continuously boosted as in perennial areas [37, 38] thus the development of the anti-disease immunity that reduces the probability of developing symptoms upon infection is delayed relative to perennial settings.

It is not certain how long protection against clinical malaria after LACT lasts, but a reasonable estimate might be between 4–8 weeks depending on the drug used, compared to 2–3 weeks for a shorter-acting ACT such as artemether-lumefantrine or artesunate-amodiaquine [12, 15, 39, 40]. These results show that the increase in the number of malaria episodes occurring between 28 and 42 days after a preceding attack was substantial in certain settings, suggesting that the additional prophylaxis will make important changes to the burden. This may be an underestimate if protection from a short-acting ACT lasts less than 28 days, or if protection from a LACT lasts longer than 6 weeks, or both. The decision to switch to ACT from non-ACT was primarily made on the basis of dramatic improvements in curative efficacy [41]. However, when choosing between the artemisinin-based combinations that are now available, all of which have similarly high curative efficacy, more focus should be given to the potential benefits other than cure.

Since long-acting ACT appears to be of most value in areas of high and seasonal transmission, there is some overlap with seasonal malaria chemoprevention (SMC), which has recently been recommended in highly seasonal areas of the Sahel and sub-Sahel with high malaria burdens [42]. SMC consists of monthly courses of sulfadoxine-pyrimethamine plus amodiaquine, and thus precludes use of two of the current ACTs: AS-SP or AS-AQ. However, the use of long-acting ACT for treatment, such as DHA-PQP or AS-MQ, may complement the SMC strategy, since a child with severe acute illness such as malaria will not receive a particular monthly course of SMC [42]. A long-acting ACT for case management would help avoid the situation where experiencing malaria once predisposes a child to experiencing malaria again later in the transmission season by preventing them being protected by SMC.

This study has a number of limitations inherent to a model-based generalization of a more complex problem. Heterogeneity in exposure to malaria is likely to be influential, since this affects how unevenly malaria is shared between individuals, with the most exposed individuals in a particular setting most likely to experience recurrent malaria in a short period of time. Similarly, acquired immunity will affect the interval between malaria episodes. Acquisition of immunity may differ in highly seasonal settings due to waning of protection during long periods without exposure, and there being some redundancy in additional infections during the transmission season among individuals who are currently or have recently been infected; both heterogeneity and acquisition of immunity are captured by the model [25], but it is not easy to capture these patterns within data in order to validate these effects. However, the consistency of the patterns of repeat malaria seen in real data and the model estimates at different levels of transmission suggest that the modelling of heterogeneity and immunity do not pose a major problem in terms of the overall interpretation. The approach used in the present analysis focused on true malaria cases that presented for treatment, which in practice may not all be treated for malaria, but also ignores use of anti-malarials for non-malaria cases, which may provide some benefits. The Markham seasonality index used in this study can underestimate the level of seasonality in areas with bimodal patterns, but the approach used here, which identified and investigated bimodality separately, suggests that areas with a high degree of bimodality are not particularly common, and that bimodality simply increases the average interval between episodes depending on the relative intensity of the two peaks and the gap between the two rainy seasons.

A limitation in scope is that the model used does not include the impact of ACT in different settings on the development of drug resistance. Long-acting ACT may be more vulnerable to development of resistance because sub-therapeutic concentrations of anti-malarials may persist for long periods after treatment, although this is not the only factor that is important [43]. Targeting long-acting ACT to areas where transmission is seasonal may help to reduce the rate at which resistance develops, by reducing the number of children who are exposed to reinfection while carrying sub-therapeutic concentrations of these drugs [44], and may help to prolong their useful life.

## Conclusion

Areas of seasonal malaria transmission are likely to have more episodes of malaria that are preventable by switching to a long-acting drug for case management than areas of equivalent transmission spread more evenly over the year. This suggests that long-acting ACT should be considered carefully as an option for case-management in areas where malaria transmission is seasonal, particularly where malaria transmission is high.

## Additional files

Additional file 1: Location of the study sites, and length of season according to Mapping Malaria Risk in Africa (MARA). Location of the six cohort/studies in West Africa, and an indication of the length of the malaria transmission season according to the Mapping Malaria Risk in Africa (MARA) project. Additional file 2: Details of the Markham Seasonality Index. Explanation of how the Markham Seasonality Index is calculated, and illustration of two example scenarios. Additional file 3: Sites with bimodal malaria transmission. Description of how sites with bimodal malaria transmission were identified, their location in Africa, an example results for 2 scenarios of bimodal transmission. Additional file 4: Markham seasonality index of clinical malaria incidence for the six studies in West Africa. Graphical representation of the Markham Seasonality Index for the 6 studies in West Africa. Additional file 5: Seasonality profiles for first administrative units at 10 % intervals of Markham Seasonality Index. Seasonality in larval carrying capacity (ability of environment to support development of mosquito larvae) for sites representing 10 % intervals of the Markham Seasonality Index. Additional file 6: Markham Seasonality Polygons for the first admin units representing 10 % intervals of Markham index. Graphical representation of the Markham Seasonality Index for the sites representing 10 % intervals of the Markham Seasonality Index. Additional file 7: Correlation between model seasonality in larval carrying capacity and predicted seasonality in clinical malaria. Correlation between Markham seasonality index calculated for the larval carrying capacity function (as an input into the model determining seasonality) and MSI for seasonality in clinical malaria at different levels of prevalence. Additional file 8: Distribution of interval between malaria episodes according to prevalence and seasonality in transmission. Distribution of intervals between malaria episodes for each of the 45 scenarios modelled. Additional file 9: Fraction of all malaria episodes that occur within 28, 42, 56 and 70 days of a previous episode. Proportion of all malaria episodes that occur within 28, 42, 56 and 70 days of a previous episode, according to seasonality and transmission intensity. Additional file 10: Total malaria burden per 1,000 children that occurs within 28, 42, 56 and 70 days of a previous episode. Total number of malaria episodes that occur within 28, 42, 56 and 70 days of a previous malaria episode, according to seasonality and transmission intensity. Additional file 11: Total malaria burden per 1,000 children in different intervals after the preceding episode. Burden of repeat malaria per 1,000 children occurring in different intervals following the previous episode, according to the Markham seasonality index (MSI). Scenarios shown for 20, 40 and 60 % prevalence. Additional file 12: Fraction of all malaria episodes in different intervals after the preceding episode. Percentage of the overall malaria burden occurring in different intervals following the previous episode, for different levels of the Markham seasonality index (MSI). Scenarios shown for 20, 40 and 60 % prevalence.

## Abbreviations

ACT: artemisinin-based combination therapy
AS-AQ: artesunate-amodiaquine
AS-MQ: artesunate-mefloquine
AS-SP: artesunate-sulfadoxine-pyrimethamine
DHA-PQ: dihydro-artemisinin-piperaquine
IBM: individual-based model
LACT: long-acting artemisinin-based combination therapy
LCC: larval carrying capacity
ITN: insecticide-treated net
MSI: Markham seasonality index
PCR: polymerase chain reaction
PTP: post-treatment prophylaxis
SMC: seasonal malaria chemoprevention
