# Supplementary material for: Seasonality in malaria transmission: implications for case-management with long-acting artemisinin combination therapy in sub-Saharan Africa
Source: Malar J. 2015 Aug 19;14:321. doi: 10.1186/s12936-015-0839-4 (PMC4539702; doi:10.1186/s12936-015-0839-4)
Supplement: Additional file 1: — Location of the study sites, and length of season according to Mapping Malaria Risk in Africa (MARA). Location of the six cohort/studies in West Africa, and an indication of the length of the malaria transmission season according to the Mapping Malaria Risk in Africa (MARA) project. [file 12936_2015_839_MOESM1_ESM.docx]

Additional File 1. Location of the study sites, and length of season according to Mapping Malaria Risk in Africa (MARA)

Location of study sites, and length of malaria transmission season according to Mapping Malaria Risk in Africa (MARA) ([www.mara.org.za](http://www.mara.org.za))
