# Supplementary material for: Seasonality in malaria transmission: implications for case-management with long-acting artemisinin combination therapy in sub-Saharan Africa
Source: Malar J. 2015 Aug 19;14:321. doi: 10.1186/s12936-015-0839-4 (PMC4539702; doi:10.1186/s12936-015-0839-4)
Supplement: Additional file 2: — Details of the Markham Seasonality Index. Explanation of how the Markham Seasonality Index is calculated, and illustration of two example scenarios. [file 12936_2015_839_MOESM2_ESM.docx]

**Additional File 2 – Details of the Markham Seasonality Index**

A seasonally varying variable in month *t* is represented by a vector of length *I­_t_* and a fixed direction θ_t_. The year (365 days) is represented by a circle of 360 degrees (2π = 6.28 radians). θ_t_ is an angle that represents the position of the midpoint of each month within the year (January 15.3°, 0.267 radians; February 44.4°, 0.775 radians; March 73.5°, 1.282 radians, etc, figure S2).

**Figure 1.. Illustration of the monthly vectors used to calculate the Markham seasonality index**

Summation of the 12 monthly vectors gives a resultant vector compared to the origin (figure S3). The length of the resultant vector as a fraction of the total length of all 12 monthly vectors gives the MSI (taking values ranging from 0, in the case where all months have equal values for the variable under consideration, and 1, in the case where the value is zero in all but one month). The direction of the resultant vector indicates when the peak in incidence occurs.

Figure S3, below, shows graphical representations of the Markham seasonality index as 2-dimensional polygons.

Formally, using the notation of Mabaso et al., 2005, the sum of the monthly vectors, *r*_k_ , is calculated as:

$$r_{k}=\sqrt{\left( \sum_{t} {\bar{I}_{t} \sin\theta_{t}} \right)^{2}+\left( \sum_{t} {\bar{I}_{t}\cos\theta_{t}} \right)^{2}}$$

And θ_k_, the direction of the resultant vector as

$\theta_{k}={tan}^{-1}\frac{\sum_{t} {\bar{I}_{t} \sin\theta_{t}}}{\sum_{t} {\bar{I}_{t} \cos\theta_{t}}}$

The Markham seasonality index (MSI) is calculated as the length of the resultant vector divided by the sum of the length of the monthly vectors.

${MSI}_{i}= \frac{r_{k}}{\sum_{t} {I_{t}}}$

**Figure 2. Graphical representation of the Markham seasonality index**

The upper plots show two hypothetical seasonality patterns. The x and y axes of the lower plots are the x and y co-ordinates specifying the location of the monthly vectors. The left-hand side shows a hypothetical scenario with no seasonality, i.e. equal values in all months. The sum of the monthly vectors returns to the origin, so the resultant vector has length zero and the MSI is therefore zero. The right-hand side shows a typical Sahelian seasonal setting, where clinical malaria incidence is concentrated between May and September; monthly vectors outside this period have no length. The length of the resultant vector (red arrow) is a high percentage of the length of the total of the monthly vectors (blue arrows), the MSI = 80.3%. The direction of the resultant vector (208.7 °, 3.64 radians) indicates that the centre of the peak occurs in July.

**References**

MABASO, M. L., CRAIG, M., VOUNATSOU, P. & SMITH, T. 2005. Towards empirical description of malaria seasonality in southern Africa: the example of Zimbabwe. Trop Med Int Health, 10, 909-18.
