# Supplementary material for: Seasonality in malaria transmission: implications for case-management with long-acting artemisinin combination therapy in sub-Saharan Africa
Source: Malar J. 2015 Aug 19;14:321. doi: 10.1186/s12936-015-0839-4 (PMC4539702; doi:10.1186/s12936-015-0839-4)
Supplement: Additional file 3: — Sites with bimodal malaria transmission. Description of how sites with bimodal malaria transmission were identified, their location in Africa, an example results for 2 scenarios of bimodal transmission. [file 12936_2015_839_MOESM3_ESM.docx]

**Additional File 3 - Sites with bimodal malaria transmission**

Because the MSI does not reliably identify sites with bimodal seasonality patterns (Mabaso et al., 2005), we used a separate approach to identify areas with bimodal seasonality and considered these separately.

**Identification of sites with bimodal transmission patterns**

We calculated the maximum fraction of annual larval carrying capacity in *n* consecutive months (with *n* ranging from 1-12 months) and compared this to the maximum fraction of annual LCC in *n* non-consecutive months (i.e. the cumulative total produced by LCC arranged in decreasing order).

Plotting the cumulative totals against the number of months gives a profile that provides an alternative approach to quantify seasonality. Non-seasonal settings (with equal incidence in each month) produce a straight line, and seasonal settings produce a steep curve (Figure 1). If the two curves diverge, this indicates a second peak in transmission distinct from the main peak. We calculated the ratio of *a*, the area between the curve for consecutive months and the line at 45° indicating equal LCC in each month to *b*, the area between the curve for non-cumulative months and the line at 45°, and used the ratio *a/b* as a measure of the degree of bimodality. A map showing how this measure varies across Africa is shown in Figure 2.

**Bimodality index for sites included in the main analyses**

As described in the paper, sites were selected at 10% intervals of the Markham seasonality index, focusing on areas without bimodal patterns which were considered separately. All sites with an MSI < 10% were strongly bimodal. For sites with MSI at 10%, 20% and 30%, there were no sites without some degree of bimodality, so the least bimodal site was chosen, these were Equateur, DRC (MSI 11.4%, bimodality ratio 0.859), Likouala, Congo (MSI 22.4%, bimodality ratio 0.930) and Western Cape, South Africa (MSI 30.7%, bimodality ratio 0.990). For all other levels of seasonality (40-90% centiles), the sites chosen were unimodal (bimodality ratio 1.00).

**Example results for bimodal sites**

Results are shown below for two examples of sites with bimodal transmission (table 1 and figure 3). The first is a case of two equally sized peaks in transmission per year (bimodality ratio 0.50). The second example, seen more frequently among the 576 first administrative areas modelled, consists of a major and minor peak at different times of the year (bimodality ratio 0.90). As might be expected, bimodality reduces the concentration of episodes in time compared to unimodal seasonal settings of similar endemicity, because there are two periods when cases are more likely to occur separated by a period of lower risk. This is particularly the case in the situation where there are two peaks of equal size, although this seasonality pattern is not particularly common.

**Definition of ‘strongly bimodal’ for main analyses**

Sites with bimodality index <0.7 were considered strongly bimodal, and excluded from the maps presented in the main paper.

**References**

MABASO, M. L., CRAIG, M., VOUNATSOU, P. & SMITH, T. 2005. Towards empirical description of malaria seasonality in southern Africa: the example of Zimbabwe. Trop Med Int Health, 10, 909-18.

Figure 1. Seasonality patterns and bimodality plots in two example bimodal sites, and one unimodal site

a) b) c)

a) Galguduud, Somalia – two equal peaks, bimodality ratio = 0.50; b) Accra, Ghana – major and minor peak, bimodality ratio=0.90; c) Nord, Cameroon, single peak, bimodality ratio=1.00. Dotted grey line indicates line of equality (i.e. assuming an equal percentage of malaria in each month of the year).

Figure 2 Map showing degree of bimodality by first administrative area

Figure shows measure of bimodality calculated as described in the text. Sites with a lower index have a stronger degree of bimodality. Sites with an index of 1 have unimodal transmission.

Table 1.Percent of repeat episodes within 28, 42, 56 and 70 days of a prior episode.

|  |  |  | % of all repeat episodes occurring within | | | |
| --- | --- | --- | --- | --- | --- | --- |
| Bimodality | Prevalence | Gini index | 28 days | 42 days | 56 days | 70 days |
| Two equal | 5% | 36.4 | 14.2 | 25.2 | 32.1 | 36.5 |
| Peaks | 10% | 38.6 | 15.7 | 26.2 | 33.3 | 37.6 |
|  | 20% | 39.6 | 16.2 | 26.6 | 34.1 | 38.5 |
|  | 40% | 43.4 | 18.0 | 29.0 | 36.3 | 41.1 |
|  | 60% | 48.3 | 20.5 | 32.8 | 40.4 | 45.5 |
|  |  |  |  |  |  |  |
| Major & minor | 5% | 51.3 | 15.4 | 26.5 | 33.9 | 42.6 |
| Peaks | 10% | 51.0 | 14.8 | 26.8 | 36.0 | 44.1 |
|  | 20% | 53.6 | 14.9 | 27.3 | 37.6 | 46.5 |
|  | 40% | 56.3 | 16.9 | 30.2 | 41.0 | 50.1 |
|  | 60% | 59.7 | 19.0 | 33.4 | 44.8 | 54.4 |

Figure 3. Model-predicted distribution of intervals between repeat episodes in example bimodal sites

a) Galguduud, Somalia – two equal peaks (top row) b) Accra, Ghana – major and minor peak (bottom row); prevalences shown left to right 5%, 10%, 20%, 40%, 60%. Colours indicate Gini index for repeat episodes: black <40%, purple <45%, navy <50%, light blue<55%, green<60%. Exact values are given in table S2.
