# Supplementary material for: Seasonality in malaria transmission: implications for case-management with long-acting artemisinin combination therapy in sub-Saharan Africa
Source: Malar J. 2015 Aug 19;14:321. doi: 10.1186/s12936-015-0839-4 (PMC4539702; doi:10.1186/s12936-015-0839-4)
Supplement: Additional file 4: — Markham seasonality index of clinical malaria incidence for the six studies in West Africa. Graphical representation of the Markham Seasonality Index for the 6 studies in West Africa. [file 12936_2015_839_MOESM4_ESM.docx]

Additional File 4. Markham seasonality index of clinical malaria incidence for the six studies in West Africa

Graphical representation of the Markham Seaonality Index for the 6 studies in West Africa. Top row Niakhar, Senegal, 2003; Farafenni, The Gambia, 2003; Kati, Mali 2008-09. Bottom row: Bousse, Burkina Faso, 2008-09; Navrongo, Ghana 2002-03; Kintampo, Ghana 2010-11. Markham seasonality indices 79.0%, 85.5%, 82.3%, 74.3%, 54.5%, 42.8%. Units of the X and Y axis are co-ordinates representing the position of the monthly vectors (based on the number of malaria cases in each month); the incidence in each month is indicated by the length of blue monthly vectors, as described in the text. The absolute values are not of major importance for interpretation; the regularity or otherwise of the shape indicates the level of seasonality (regular dodecagon: non-seasonal, crescent shapes = seasonal).
