# Supplementary material for: Seasonality in malaria transmission: implications for case-management with long-acting artemisinin combination therapy in sub-Saharan Africa
Source: Malar J. 2015 Aug 19;14:321. doi: 10.1186/s12936-015-0839-4 (PMC4539702; doi:10.1186/s12936-015-0839-4)
Supplement: Additional file 5: — Seasonality profiles for first administrative units at 10 % intervals of Markham Seasonality Index. Seasonality in larval carrying capacity (ability of environment to support development of mosquito larvae) for sites representing 10 % intervals of the Markham Seasonality Index. [file 12936_2015_839_MOESM5_ESM.docx]

Additional File 5. Seasonality profiles for first administrative units at 10% intervals of Markham Seasonality Index

Seasonality in larval carrying capacity (ability of environment to support development of mosquito larvae) for sites representing 10% intervals of the Markham Seasonality Index. Top row (10%, 20%, 30%), middle row (40%, 50%, 60%), bottom row (70%, 80%, 90%). For clarity of presentation two annual cycles are shown. For more details on larval carrying capacity, see Griffin et al. PLoS Med, 2010 doi:10.1371/journal.pmed.1000324.
