# Supplementary material for: Seasonality in malaria transmission: implications for case-management with long-acting artemisinin combination therapy in sub-Saharan Africa
Source: Malar J. 2015 Aug 19;14:321. doi: 10.1186/s12936-015-0839-4 (PMC4539702; doi:10.1186/s12936-015-0839-4)
Supplement: Additional file 6: — Markham Seasonality Polygons for the first admin units representing 10 % intervals of Markham index. Graphical representation of the Markham Seasonality Index for the sites representing 10 % intervals of the Markham Seasonality Index. [file 12936_2015_839_MOESM6_ESM.docx]

Additional File 6. Markham Seasonality Polygons for the first admin units representing 10% intervals of Markham index.

Seasonality patterns for sites representing 10% intervals of the Markham Seasonality Index. Top row (10%, 20%, 30%), middle row (40%, 50%, 60%), bottom row (70%, 80%, 90%). Units of the X and Y axis are co-ordinates representing the position of the monthly vectors (based on the number of malaria cases in each month): the incidence in each month is indicated by the length of blue monthly vectors, as described in the text. Sites with relatively little seasonality (top row) result in a regular shape and a small resultant vector relative to the length of all the monthly vectors (MSI close to 0). Sites where malaria transmission is concentrated in a few months of the year (bottom row) result in an asymmetric shape and a long resultant vector relative to the monthly vectors (MSI closer to 1).
