# Supplementary material for: Seasonality in malaria transmission: implications for case-management with long-acting artemisinin combination therapy in sub-Saharan Africa
Source: Malar J. 2015 Aug 19;14:321. doi: 10.1186/s12936-015-0839-4 (PMC4539702; doi:10.1186/s12936-015-0839-4)
Supplement: Additional file 7: — Correlation between model seasonality in larval carrying capacity and predicted seasonality in clinical malaria. Correlation between Markham seasonality index calculated for the larval carrying capacity function (as an input into the model determining seasonality) and MSI for seasonality in clinical malaria at different levels of prevalence. [file 12936_2015_839_MOESM7_ESM.docx]

Additional File 7. Correlation between model seasonality in larval carrying capacity and predicted seasonality in clinical malaria

Markham seasonality index calculated for the larval carrying capacity function (as an input into the model determining seasonality) and seasonality in clinical malaria at different levels of prevalence. This shows that seasonality in the input is reflected in the malaria incidence patterns, although as might be expected, clinical incidence is slightly less seasonal than the suitability of the environment for transmission.
