# Supplementary material for: Seasonality in malaria transmission: implications for case-management with long-acting artemisinin combination therapy in sub-Saharan Africa
Source: Malar J. 2015 Aug 19;14:321. doi: 10.1186/s12936-015-0839-4 (PMC4539702; doi:10.1186/s12936-015-0839-4)
Supplement: Additional file 8: — Distribution of interval between malaria episodes according to prevalence and seasonality in transmission. Distribution of intervals between malaria episodes for each of the 45 scenarios modelled. [file 12936_2015_839_MOESM8_ESM.docx]

Additional File 8. Distribution of interval between malaria episodes according to prevalence and seasonality in transmission

Distribution of intervals between malaria episodes for each of the 45 scenarios modelled. The rows show different levels of prevalence (5%, 10%, 20%, 40%, 60%) and the columns show different levels of seasonality (Markham seasonality index (MSI) from left to right of 10%, 20%, 30%, 40%, 50%, 60%, 70%, 80%, and 90%).
