# Supplementary material for: Seasonality in malaria transmission: implications for case-management with long-acting artemisinin combination therapy in sub-Saharan Africa
Source: Malar J. 2015 Aug 19;14:321. doi: 10.1186/s12936-015-0839-4 (PMC4539702; doi:10.1186/s12936-015-0839-4)
Supplement: Additional file 9: — Fraction of all malaria episodes that occur within 28, 42, 56 and 70 days of a previous episode. Proportion of all malaria episodes that occur within 28, 42, 56 and 70 days of a previous episode, according to seasonality and transmission intensity. [file 12936_2015_839_MOESM9_ESM.docx]

Additional File 9. Fraction of all malaria episodes that occur within 28, 42, 56 and 70 days of a previous episode

Proportion of all malaria episodes that occur within 28, 42, 56 and 70 days of a previous episode, according to seasonality and transmission intensity.
