# Supplementary material for: Seasonality in malaria transmission: implications for case-management with long-acting artemisinin combination therapy in sub-Saharan Africa
Source: Malar J. 2015 Aug 19;14:321. doi: 10.1186/s12936-015-0839-4 (PMC4539702; doi:10.1186/s12936-015-0839-4)
Supplement: Additional file 10: — Total malaria burden per 1,000 children that occurs within 28, 42, 56 and 70 days of a previous episode. Total number of malaria episodes that occur within 28, 42, 56 and 70 days of a previous malaria episode, according to seasonality and transmission intensity. [file 12936_2015_839_MOESM10_ESM.docx]

Additional File 10. Total malaria burden per 1000 children that occurs within 28, 42, 56 and 70 days of a previous episode

Total number of malaria episodes that occur within 28, 42, 56 and 70 days of a previous malaria episode, according to seasonality and transmission intensity.
