# Supplementary material for: Seasonality in malaria transmission: implications for case-management with long-acting artemisinin combination therapy in sub-Saharan Africa
Source: Malar J. 2015 Aug 19;14:321. doi: 10.1186/s12936-015-0839-4 (PMC4539702; doi:10.1186/s12936-015-0839-4)
Supplement: Additional file 11: — Total malaria burden per 1,000 children in different intervals after the preceding episode. Burden of repeat malaria per 1,000 children occurring in different intervals following the previous episode, according to the Markham seasonality index (MSI). Scenarios shown for 20, 40 and 60 % prevalence. [file 12936_2015_839_MOESM11_ESM.docx]

Additional File 11. Total malaria burden per 1000 children in different intervals after the preceding episode

Burden of repeat malaria per 1000 children occurring in the intervals shown, for different levels of the Markham seasonality index (MSI). A) prevalence in 2 to 10-year-old children, 20%; B) prevalence in 2 to 10-year-old children, 40%; C) prevalence in 2 to 10-year-old children, 60%.
