# Supplementary material for: Seasonality in malaria transmission: implications for case-management with long-acting artemisinin combination therapy in sub-Saharan Africa
Source: Malar J. 2015 Aug 19;14:321. doi: 10.1186/s12936-015-0839-4 (PMC4539702; doi:10.1186/s12936-015-0839-4)
Supplement: Additional file 12: — Fraction of all malaria episodes in different intervals after the preceding episode. Percentage of the overall malaria burden occurring in different intervals following the previous episode, for different levels of the Markham seasonality index (MSI). Scenarios shown for 20, 40 and 60 % prevalence. [file 12936_2015_839_MOESM12_ESM.docx]

Additional File 12. Fraction of all malaria episodes in different intervals after the preceding episode

Percentage of the overall malaria burden occurring after a previous episode in the intervals shown, for different levels of the Markham seasonality index (MSI). A) prevalence in 2 to 10-year-old children, 20%; B) prevalence in 2 to 10-year-old children, 40%; C) prevalence in 2 to 10-year-old children, 60%.
